# Association Between Educational Attainment and Depressive Symptom Severity Among Young-Old Adults Aged 60–74 Years: The Mediating Role of Self-Reported Memory Function

**DOI:** 10.3390/bs16081264

**Published:** 2026-07-23

**Authors:** Beifen Pan, Pu Ge, Ruge Liu, Yangyun Ou, Junchen Jiang, Yulin Wang, Kaiqiang Dong, Min Wang, Dong Zhang, Lixian Cui, Rongjuan Guo

**Affiliations:** 1 The Second Clinical College, Beijing University of Chinese Medicine, Beijing 100078, China; wwwpbf19980828@163.com (B.P.);; 2 School of Traditional Chinese Medicine, Beijing University of Chinese Medicine, Beijing 100029, China; 17853140673@163.com

**Keywords:** young-old adults, educational attainment, depressive symptom severity, self-reported memory function, mediation effect

## Abstract

**Background:** This study aimed to investigate the association between educational attainment and depressive symptoms among young-old adults aged 60–74 years, and to examine the mediating effect of self-reported memory function between them, so as to provide a reference for the early screening and intervention of depressive symptom severity in this population. **Methods**: This was a cross-sectional study, and data were derived from the 2022 China Family Panel Studies (CFPS). After data cleaning, 3666 valid samples were included. Depressive symptom severity was assessed using the 8-item Center for Epidemiologic Studies Depression Scale (CES-D 8) (Cronbach’s α = 0.771, Guttman Split-Half Coefficient = 0.742), with a total score ≥ 9 defined as probable depression. Self-reported memory function was measured using a single item (scored 0–4), with higher scores indicating better perceived memory, and was treated as a continuous variable for analysis. Descriptive statistics and Pearson correlation analysis were performed using R software (version 4.2.3). The mediation effect was tested using the product-of-coefficients method combined with the bootstrap method (5000 resamples), supplemented by a quasi-Bayesian model and multiple sensitivity analyses (including outcome variable substitution and model diagnostic corrections) for robustness testing. **Results**: Among the study participants, the prevalence of probable depression was 25.18%. Correlation analyses showed that educational attainment was significantly negatively correlated with depressive symptom severity (*r* = −0.177, *p* < 0.001) and significantly positively correlated with self-reported memory function (*r* = 0.241, *p* < 0.001). Self-reported memory function was significantly negatively correlated with depressive symptom severity (*r* = −0.223, *p* < 0.001). After adjusting for age, gender, place of residence, subjective income, self-rated health, and chronic disease status, mediation analysis showed that the direct effect of educational attainment on depressive symptom severity was −0.297 (95% CI: −0.424 to −0.174), while the indirect effect via self-reported memory function was −0.109 (95% CI: −0.139 to −0.082). The direct and indirect effects accounted for 73.20% and 26.80% of the total effect, respectively; the results of the quasi-Bayesian analysis and multiple sensitivity analyses were consistent in direction, further validating the stability of this mediation effect. **Conclusions**: Among young-old adults aged 60–74 years, self-reported memory function plays a partial statistical mediating role in the association between educational attainment and depressive symptom severity. This suggests that strengthening attention to memory status and early screening among individuals with lower educational attainment may offer a potential intervention approach for alleviating depressive symptom severity in this population in the future.

## 1. Introduction

Depression is a common emotional and mental health condition characterized by persistent low mood and anhedonia, and it is associated with a high incidence, high recurrence rate, and substantial social and health burden (Miret et al., 2013). Individuals experiencing depression often present with multisystem manifestations, including sleep disturbances, weight fluctuations, appetite changes, and cognitive impairment, which severely compromise their quality of life and social functioning (Malhi & Mann, 2018). It should be noted that even in the absence of a clinical diagnosis, subthreshold depressive symptoms remain highly prevalent among older adults (X. Zhao et al., 2024b), and as global population aging accelerates, depressive symptoms in older adults have become a prominent public health issue (Cai et al., 2023). Young-old adults aged 60–74 years are undergoing role transitions such as retirement (Mosconi et al., 2023), accompanied by progressive changes in physical function. The risk of depressive symptoms during this life stage is closely associated with physical functional status (Zhang et al., 2025); therefore, this population represents a key research focus in geriatric mental health. The rationale for focusing on adults aged 60–74 years rather than the oldest-old is detailed in Section 2.1. With respect to cognitive health risk, epidemiological data from mainland China indicate that the prevalence of dementia is approximately 3.6% among adults aged 70–74 years but rises sharply to 5.9% among those aged 75–79 years (Y. T. Wu et al., 2013), suggesting that age 75 may represent a critical juncture at which the risk structure of cognitive function in older adults undergoes a transition. This pattern provides indirect support for using age 75 as the age cutoff in the present study.

Cognitive dysfunction and depressive symptoms are closely associated, as demonstrated by numerous studies: cognitive impairment (such as memory decline, reduced attention, and diminished executive function) is both a common concomitant manifestation of depressive symptoms and an important risk factor for them (S. Li et al., 2025b; Bortolato et al., 2014; Ma et al., 2025; Murrough et al., 2011). For instance, a longitudinal study of middle-aged and older Chinese adults demonstrated that early cognitive function (particularly memory and orientation) significantly predicted subsequent depressive symptoms, providing direct evidence for the hypothesis that cognitive impairment serves as a crucial vulnerability factor for late-life depressive symptoms, with this association being more pronounced among women (Ma et al., 2025). Furthermore, cognitive function not only affects the risk of developing depressive symptoms but is also closely associated with intervention outcomes. Research indicates that poorer cognitive function (especially semantic fluency within executive function) reduces an individual’s adherence to treatment and compromises the completion of standard antidepressant therapy (Cristancho et al., 2018). This body of evidence suggests that cognitive factors play multiple roles in the occurrence, progression, and treatment outcomes of late-life depressive symptoms. However, the specific effect of cognitive decline as a risk factor for depressive symptoms remains controversial, with inconsistent findings across different studies (C. Li et al., 2025a). This inconsistency hints at the potential existence of more complex mediating or moderating mechanisms between the two.

Among the various dimensions of cognitive function, self-reported memory function—defined as an individual’s subjective perception and evaluation of their everyday memory performance—is a core indicator within the framework of Subjective Cognitive Decline (SCD) (Jessen et al., 2014). Owing to its ability to closely reflect real-life situations, provide earlier indications of subjective cognitive changes, and allow for practical measurement in large-scale surveys, it has become a key indicator in research on cognition and emotion in older adults (Huang & Maurer, 2019; Rickenbach et al., 2015). It should be noted that self-reported memory function, as a subjective cognitive evaluation, is influenced not only by an individual’s objective cognitive status but also by their concurrent emotional state, with both factors potentially jointly shaping respondents’ self-reports (Brailean et al., 2019). Previous studies have shown that older adults who report poorer memory function are more likely to experience loneliness, helplessness, and hopelessness, which may in turn increase their risk of depressive symptoms. In contrast, better self-reported memory function may help preserve older adults’ sense of self-efficacy and promote positive emotional well-being (Brailean et al., 2019).

Meanwhile, educational attainment has been widely recognized as a significant protective factor against late-life depressive symptoms (J. Li et al., 2026; Torres et al., 2019; Xin & Ren, 2020). However, existing research predominantly focuses on the direct effect of educational attainment on depressive symptoms or examines the overall mediating role of general cognitive function. There is a paucity of studies that delve into the specific mediating pathways of core cognitive components—particularly self-reported memory function as a key dimension of cognition. Whether self-reported memory function plays a bridging role between educational attainment and late-life depressive symptoms remains insufficiently explored.

Based on the cognitive reserve theory, lifelong experiences such as education can build cognitive reserve, enhancing an individual’s resilience to neuropathological changes and functional maintenance capacity (Stern, 2012). For young-old adults, a higher educational attainment implies richer early-life cognitive stimulation. This reserve not only delays objective cognitive decline but also maintains individuals’ positive perception of their daily memory capacity (i.e., self-reported memory function) (Clouston et al., 2020). Conversely, insufficient cognitive reserve resulting from lower educational attainment may accelerate an individual’s subjective cognitive decline, thereby increasing the risk of mental health issues such as depressive symptoms (Brailean et al., 2019). Recent studies further confirm that cognitive decline is significantly associated with worsening depressive symptoms in older adults, suggesting the critical role of cognitive function in the development of depressive symptoms (Yang et al., 2023). Accordingly, we hypothesize that higher educational attainment may be associated with lower depressive symptom severity among young-old adults, partly through its association with better self-reported memory function. Although a recent study explored the parallel mediating effects of economic status and subjective memory across the entire older population (R. Zhao et al., 2024a), two critical limitations remain. First, cognitive decline in the oldest-old population may be confounded by a higher prevalence of organic pathologies and severe somatic diseases, thereby failing to focus on the young-old population aged 60–74, who present better intervention efficacy and more homogeneous daily self-care abilities. Second, prior research has largely relied on a single frequentist statistical approach, lacking cross-validation for the robustness of the mediation pathway.

Therefore, this study utilized the most recent 2022 CFPS data to focus on young-old adults aged 60–74 years, with particular emphasis on examining the mediating role of self-reported memory function—a core indicator—in the association between educational attainment and depressive symptoms. A quasi-Bayesian sensitivity analysis was further introduced to cross-validate the robustness of the mediation effect, in order to address the aforementioned limitations of previous research and provide more targeted empirical reference for the screening of cognition-related depressive symptoms in this population.

## 2. Data and Methods

### 2.1. Study Participants and Data Source

The data for this study were obtained from the 2022 China Family Panel Studies (CFPS), implemented by the Institute of Social Science Survey of Peking University from May to December 2022. The CFPS is a nationwide, large-scale, and multidisciplinary longitudinal tracking survey. Utilizing a multi-stage stratified probability proportional to size (PPS) sampling method, the sample covers 25 provinces, municipalities, and autonomous regions in China, thereby ensuring good national representativeness. The analysis of this study was conducted based on the original sampling framework of the CFPS, demonstrating good potential for external generalizability.

Initially, a total of 5967 older respondents aged 60–79 from the CFPS database were included in this study. To ensure the quality of the analysis, rigorous data cleaning was performed, resulting in the exclusion of 1851 cases: (1) Given that the completeness of core variable measurement is critical for mediation model estimation, this study did not use multiple imputation; instead, listwise deletion was applied to exclude cases with missing values, non-applicable answers, or invalid responses among the covariates (age, sex, residence, subjective income, self-reported health, and chronic diseases) and core research variables (educational attainment, depressive symptom severity, and self-reported memory function). This approach may introduce a degree of sample selection bias, which is discussed further in the limitations section; (2) based on the questionnaire definitions and logic, cases with logical contradictions or clearly abnormal values were eliminated.

Building on the data cleaning process, this study further excluded 450 older adults aged 75–79, ultimately focusing on the young-old population aged 60–74. This selection was primarily intended to ensure homogeneity in social participation and daily self-care ability within the sample, so as to more accurately assess the statistical mediating role of self-reported memory function in the association between educational attainment and depressive symptom severity, while avoiding excessive interference from the higher prevalence of physical illnesses in older age groups.

**Figure 1:**
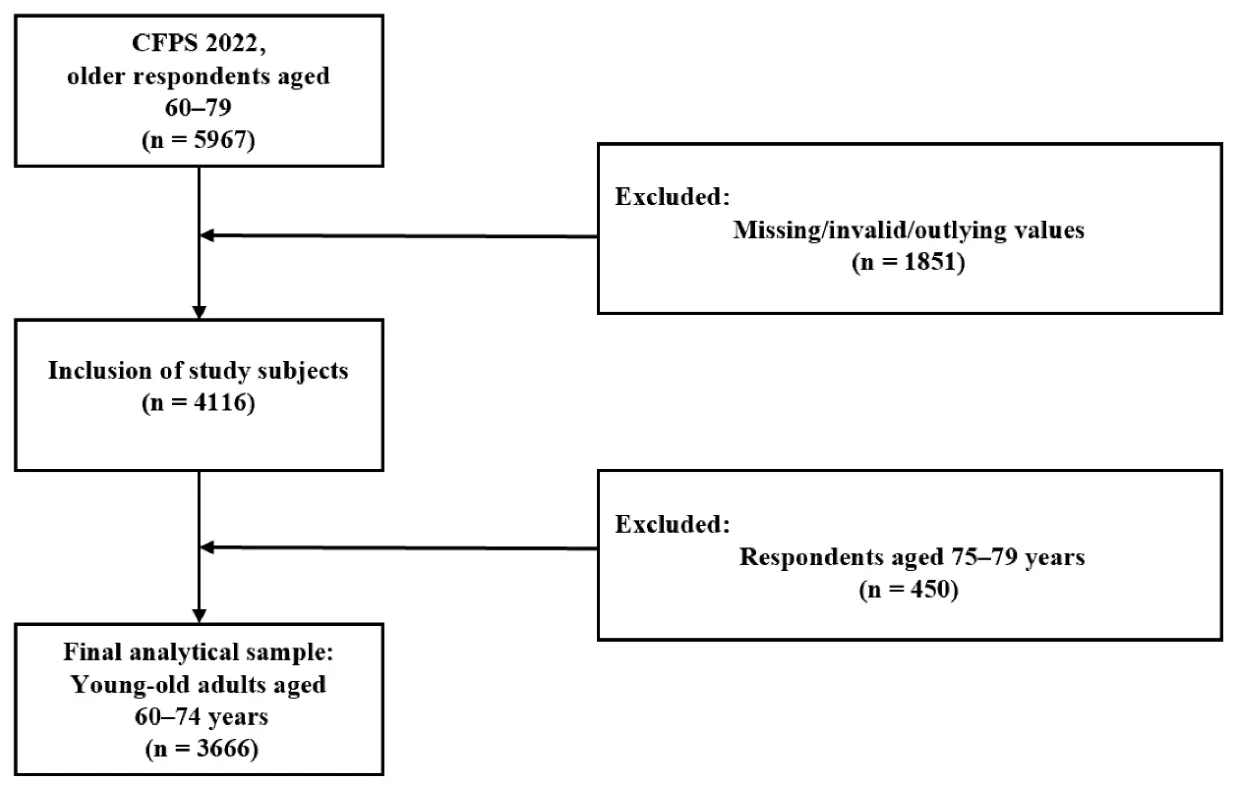
Flowchart of Research Subject Selection Process. (Note: The initial sample included 5967 older adults aged 60–79 years from the 2022 CFPS database. After sequentially excluding (1) cases with missing, invalid, or outlying values in core study variables and covariates, and (2) respondents aged 75–79 years, 3666 valid participants aged 60–74 years were included in the final analysis. Core study variables: Educational attainment, depressive symptom severity, self-reported memory function. Covariates: age, sex, residence, subjective income, self-rated health, and chronic disease status).

After the aforementioned screening, a final effective sample of 3666 young-old adults aged 60–74 was obtained. This study is a secondary analysis based on publicly available CFPS data, and the final sample size was determined by the cases in the 2022 wave of the CFPS that met the inclusion criteria and had complete data. No a priori sample size calculation was performed; however, all eligible participants were included to maximize statistical power. The detailed flowchart of the participant selection process is shown in Figure 1.

### 2.2. Research Variables and Measurement Tools

Data for all variables in this study were derived from the standardized questionnaire of the 2022 CFPS. The definitions, coding, and measurement methods for each variable are detailed below.

#### 2.2.1. Covariates (Control Variables)

To control for the effects of potential confounding factors, demographic and health-related variables were selected as covariates (control variables) in this study. Age was a continuous variable retained at its original CFPS survey value, while the remaining categorical covariates were reclassified and converted into binary (dichotomous) codes, with specific processing and coding as follows: Sex was coded as a dichotomous variable (0 = female, 1 = male). Residence was coded as a dichotomous variable (0 = rural, 1 = urban). Subjective income level was reclassified based on the original 1–5 scale: scores of 1–2 were defined as a lower level (coded as 0), and scores of 3–5 as a higher level (coded as 1). Self-rated health status was reclassified based on the original 0–4 scale: scores of 0–1 (poor/fair) were defined as poor (coded as 0), and scores of 2–4 (good/very good/excellent) were defined as good (coded as 1). Chronic disease status was a dichotomous variable (0 = no history of chronic disease, 1 = history of chronic disease). These variables have all been confirmed as core influencing factors of depressive symptoms in older adults in previous studies (S. Li et al., 2025b; Xin & Ren, 2020) and were therefore included as control variables in the statistical mediation analysis of this study.

#### 2.2.2. Independent Variable: Educational Attainment

In this study, educational attainment was assigned rank scores based on the original CFPS survey results and treated as a continuous variable. The specific assignments were as follows: 0 = no formal schooling and illiterate/semi-illiterate, 1 = primary school, 2 = junior high school, 3 = senior high school/technical secondary school/technical school/vocational high school, and 4 = junior college/bachelor’s degree/master’s degree. Notably, although educational attainment is inherently an ordinal categorical variable, it was analyzed as a continuous variable in this study. Methodological simulation research has demonstrated that when an ordinal variable contains five or more categories, treating it as a continuous variable in statistical models introduces negligible bias and yields robust results (Bollen & Barb, 1981), which aligns with the conventional handling of ordinal variables in mediation analysis (Rhemtulla et al., 2012). In addition, to further examine the robustness of this treatment, this study also conducted a Spearman correlation analysis and a sensitivity analysis treating educational attainment as a categorical variable; the results were consistent with those of the main analysis (see Supplementary Tables S1 and S2).

#### 2.2.3. Dependent Variable: Depressive Symptom Severity

Depressive symptom severity was assessed using the 8-item Center for Epidemiologic Studies Depression Scale (CES-D 8). This scale is suitable for rapid screening of depressive symptoms in community-dwelling older adults and includes four dimensions: somatic symptoms, interpersonal relationships, positive affect, and depressed affect (Turvey et al., 1999). Respondents were asked to self-report the frequency of each corresponding emotion/behavior over the past week using a 4-point Likert scale: 0 = rarely or none of the time (<1 day), 1 = some or a little of the time (1–2 days), 2 = occasionally or a moderate amount of time (3–4 days), and 3 = most or all of the time (5–7 days). The specific items were: D1 (I felt depressed), D2 (I felt that everything I did was an effort), D3 (My sleep was restless), D4 (I was happy), D5 (I felt lonely), D6 (I enjoyed life), D7 (I felt sad), and D8 (I could not get “going”). Items D4 and D6 were reverse-scored using the following rule: 3→0, 2→1, 1→2, 0→3. After reverse-scoring, the scores for all eight items were summed to obtain a total depressive symptom score, ranging from 0 to 24, with higher scores indicating greater depressive symptom severity. In this study, the scale demonstrated good internal consistency (Cronbach’s α = 0.771) and split-half reliability (Guttman Split-Half Coefficient = 0.742). Following previous research standards (Briggs et al., 2018), a total score of ≥9 was defined as the presence of probable depression. In this study, the CES-D8 was used to assess depressive symptom severity and probable depression, rather than clinically diagnosed depressive disorders.

#### 2.2.4. Mediator: Self-Reported Memory Function

Self-reported memory function was measured using a single standardized item (Q501) from the 2022 CFPS questionnaire. The question was: “How much of the main things that happened to you in the past week can you remember?” The original response was a 5-point scale ranging from 0 to 4, with higher scores indicating a stronger self-perceived ability to remember daily events. The specific scoring rules were as follows: 0 = Can remember only a little bit (also scored 0 if the respondent indicated they could remember nothing), 1 = Can remember only a few, 2 = Can remember about half, 3 = Can remember most of it, 4 = Can remember all of it. For the mediation analysis, this original 0–4 score was used as a continuous variable. It should be noted that, as a single self-reported item, although this measure offers high feasibility for implementation in large-scale epidemiological surveys, its construct coverage is relatively limited, and self-reported evaluations are susceptible to mood-congruent bias (Brailean et al., 2019); this issue will be further discussed in the limitations section.

#### 2.2.5. Statistical Analysis

Data analysis was performed using R software (version 4.2.3), with a two-tailed significance level set at α = 0.05. First, a descriptive analysis was conducted on the basic characteristics of the study participants, where categorical variables were expressed as frequencies and percentages [*n* (%)], and continuous variables were presented as means ± standard deviations ($\bar{x}$ ± s). The internal consistency of the CES-D8 scale was evaluated using Cronbach’s α and split-half reliability. Pearson correlation analysis was employed to preliminarily explore the bivariate relationships among educational attainment, self-reported memory function, and depressive symptom severity.

The primary mediation analysis was conducted within a linear regression framework: (1) a total effect model was established to examine the total effect of educational attainment on depressive symptom severity; (2) a Path *a* model was established to examine the predictive effect of educational attainment on self-reported memory function; and (3) a direct effect model was established, simultaneously including educational attainment and self-reported memory function, to estimate Path *b* (the effect of self-reported memory function on depressive symptom severity) as well as the direct effect of educational attainment. The indirect effect was calculated using the product-of-coefficients method (*a* × *b*), and based on these regression coefficients, the bootstrap method (with 5000 resamples) was employed to estimate the 95% confidence interval (CI). All of the aforementioned models adjusted for age, gender, place of residence, subjective income, self-rated health, and chronic disease status. Given the limitations of the cross-sectional design, the findings of this study were interpreted solely as statistical mediation or indirect associations, without making causal inferences.

To further examine the statistical assumptions and robustness of the results, the following supplementary analyses were conducted: multicollinearity was assessed using the variance inflation factor (VIF); heteroscedasticity was assessed using the Breusch-Pagan test; and residual and influential-point diagnostics were performed using residual plots and Q-Q plots (see Supplementary Figures S1 and S2) together with Cook’s distance. The model was also re-estimated using HC3 robust standard errors (see Supplementary Table S4). Given the ordinal nature of educational attainment and self-reported memory function, in addition to the aforementioned Spearman correlation analysis and the sensitivity analysis treating educational attainment as a categorical variable (see Supplementary Table S1 and S2), this study further defined “probable depression” as CES-D8 ≥ 9 and re-estimated the mediation pathway using logistic regression and modified Poisson regression with robust standard errors (bootstrap, 5000 resamples; see Supplementary Table S3). In addition, a quasi-Bayesian approach with 1000 Monte Carlo simulations was employed to cross-validate the stability of the mediation effect under different statistical assumptions (see Section 2.2.6). To evaluate the potential selection bias introduced by listwise deletion, this study further compared, within the 60–79 age range, the age and sex distributions of the final analytic sample (*n* = 3666) with those of the 2301 excluded cases—comprising 1851 cases excluded due to missing core variables and 450 cases excluded due to older age (75–79 years) (see Supplementary Table S5).

#### 2.2.6. Quasi-Bayesian Robustness Check

To further verify the robustness of the estimated indirect effect, a quasi-Bayesian approximation approach was employed to re-estimate the model in this study. In terms of prior specification, no prior distributions were explicitly specified because this procedure was a quasi-Bayesian approximation rather than a fully Bayesian model. Based on the asymptotic normality of frequentist regression coefficients, parameters were drawn from a multivariate normal distribution through 1000 Monte Carlo simulations, thereby generating a simulated distribution of the indirect effect and calculating the 95% quasi-Bayesian confidence interval. Compared with methods that rely on point estimates of ordinary least squares (OLS) parameters and their conventional hypothesis tests (e.g., the Sobel test), the quasi-Bayesian approach does not depend on the stringent assumption that the product of the indirect effect (a × b) must strictly follow a normal distribution, thereby addressing the potential non-normality of the product distribution. If this interval does not contain 0, it indicates that the estimated indirect effect is statistically significant and provides additional support for the robustness of the estimated indirect association.

## 3. Results

A total of 3666 eligible young-old adults aged 60–74 were included in this study. The screening process and the details of sample exclusion at each stage are illustrated in Figure 1.

### 3.1. Basic Characteristics of the Study Participants

The general characteristics of the participants and the descriptive statistics of the core variables are presented in Table 1 and Table 2.

Categorical variables (*n* = 3666) are presented as counts (percentages) [*n* (%)]. The sample consisted of 1915 males (52.24%) and 1751 females (47.76%); 1873 individuals residing in rural areas (51.09%) and 1793 in urban areas (48.91%); 2712 participants with a higher subjective income level (73.98%) and 954 with a lower level (26.02%); 2261 participants with good self-rated health (61.67%) and 1405 with poor self-rated health (38.33%); and 2541 without chronic diseases (69.31%) and 1125 with the presence of chronic disease(s) (30.69%).

Continuous variables are presented as means ± standard deviations (M ± SD): age was 66.40 ± 4.105 years, educational attainment (range: 0–4) was 1.36 ± 1.191, self-reported memory function (range: 0–4) was 1.60 ± 1.301, and depressive symptom severity (CES-D 8 total score, range: 0–24) was 5.80 ± 4.483 (higher scores indicate more severe depressive symptoms).

### 3.2. Distribution of CES-D8 Item Scores and Probable Depression Status

The assessment of depressive status among the 3666 respondents showed that 2743 individuals (74.82%) did not meet the criteria for probable depression (CES-D 8 total score < 9), while 923 individuals (25.18%) were classified as having probable depression (CES-D 8 total score ≥ 9). The mean scores for the eight CES-D8 items ranged from 0.33 (Item D8: “I could not get ‘going’“) to 0.97 (Item D3: “My sleep was restless”), with Item D3 having the highest mean score. Regarding the distribution of scores across each item, Item D8 had the highest percentage of respondents scoring 0 (78.31%) and the lowest percentage scoring 3 (4.09%). The detailed mean scores and distribution for each item level are presented in Table 3.

### 3.3. Correlations Among Educational Attainment, Self-Reported Memory Function, and Depressive Symptom Severity

The results of the Pearson correlation analysis are presented in Table 4. The analysis revealed significant bivariate correlations among educational attainment, self-reported memory function, and depressive symptom severity (CES-D8 total score) in young-old adults aged 60–74 years (all *p* < 0.001). Specifically: (1) Educational attainment was significantly negatively correlated with depressive symptom severity (*r* = −0.177), indicating that higher education was associated with milder depressive symptoms; (2) Educational attainment was significantly positively correlated with self-reported memory function (*r* = 0.241), suggesting that higher education was linked to better self-reported memory; (3) Self-reported memory function was significantly negatively correlated with depressive symptom severity (*r* = −0.223), meaning that better self-reported memory was associated with less severe depressive symptoms.

### 3.4. Statistical Mediation Analysis of Self-Reported Memory Function

After controlling for age, sex, residence, subjective income, self-rated health, and chronic disease status, the results of the mediation analysis regarding the role of self-reported memory function between educational attainment and depressive symptom severity are presented below: (1) In Model 1 (with depressive symptom severity as the dependent variable), educational attainment was significantly negatively associated with depressive symptom severity (B = −0.407, *p* < 0.001), with a model R^2^ of 0.153 (adjusted R^2^ = 0.151). (2) In Model 2 (with self-reported memory function as the dependent variable), educational attainment was significantly positively associated with self-reported memory function (B = 0.221, *p* < 0.001), with a model R^2^ of 0.085 (adjusted R^2^ = 0.083). (3) In Model 3 (dependent variable: depressive symptom severity, incorporating the mediator), both educational attainment (B = −0.297, *p* < 0.001) and self-reported memory function (B = −0.495, *p* < 0.001) were significantly negatively associated with depressive symptom severity. The model R^2^ increased to 0.172 (adjusted R^2^ = 0.170), and the absolute value of the partial regression coefficient for educational attainment decreased, suggesting that self-reported memory function plays a partial statistical mediating role between educational attainment and depressive symptom severity (see Table 5 for details). Building upon the primary mediation analysis, a sensitivity analysis was further conducted in this study (detailed in Section 2.2.6 Robustness Check).

### 3.5. Bootstrap Statistical Mediation Test of Self-Reported Memory Function

**Figure 2:**
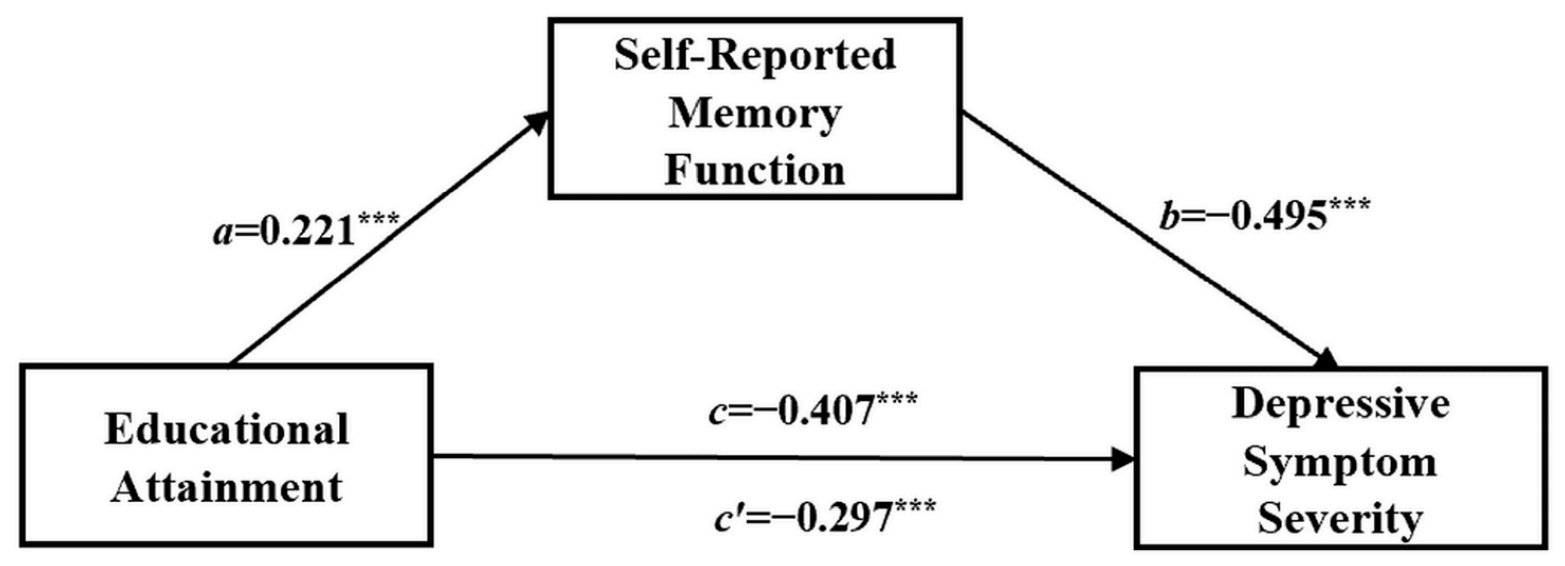
Path diagram of the mediating effect of Self-Reported Memory Function between Educational Attainment and Depressive Symptom Severity (for young-old adults aged 60–74 years). *** *p* < 0.001; Paths a, b, c, and c’ are detailed in Table 5 and Table 6.

In this study, a bias-corrected percentile bootstrap method (with 5000 resamples) was used to test the significance of the mediation effect and calculate the 95% confidence intervals (CIs). The results showed that the direct effect of educational attainment on depressive symptom severity was −0.297 (95% CI: −0.424 to -0.174, *p* < 0.001), and the indirect effect via self-reported memory function was −0.109 (95% CI: −0.139 to −0.082). Since the interval did not contain 0, it indicated that the indirect effect was statistically significant. Further calculation of the effect proportions revealed that the direct and indirect effects accounted for 73.20% and 26.80% of the total effect, respectively. This demonstrates that self-reported memory function plays a partial statistical mediating role in the association between educational attainment and depressive symptom severity among young-old adults aged 60–74 years. Detailed path coefficients and test results are presented in Figure 2 and Table 6.

### 3.6. Results of the Robustness Check

The results of the quasi-Bayesian mediation analysis showed that, after adjusting for covariates including sex, residence, subjective income, self-rated health, chronic disease status, and age, educational attainment remained significantly and positively associated with self-reported memory function (*B* = 0.221, *p* < 0.001); the total effect of educational attainment on depressive symptom severity total score was significant (*B* = −0.407, *p* < 0.001). Further decomposition of the effects (Table 7) revealed an estimated indirect effect of −0.109 (95% CI: −0.140 to −0.081, *p* < 0.001) and a direct effect of −0.297 (95% CI: −0.423 to −0.175, *p* < 0.001). The mediation proportion was approximately 26.80%. These results are highly consistent with the aforementioned Bootstrap test findings, confirming the robust nature of the partial statistical mediating role of self-reported memory function between educational attainment and depressive symptom severity.

## 4. Discussion

This study, utilizing a nationally representative sample from the 2022 CFPS, systematically explored the associations and underlying mechanisms among educational attainment, self-reported memory function, and depressive symptom severity in young-old adults aged 60–74 years. Results showed that educational attainment was significantly negatively correlated with depressive symptom severity, and self-reported memory function played a partial mediating role, accounting for 26.80% of this association, suggesting that educational attainment was directly associated with lower depressive symptom severity among young-old adults and may also be indirectly associated with it through maintaining or enhancing self-reported memory function. This result is consistent with the conclusions of previous studies conducted among older populations (Chang-Quan et al., 2010). This is precisely the core incremental contribution of this study relative to the existing literature: compared with a recent similar study (R. Zhao et al., 2024a), this study focused strictly on the 60–74 age group, a more homogeneous young-old population, and concentrated on self-reported memory function as a single cognitive mediating pathway (without incorporating parallel mediators such as economic factors). Building on the conventional bootstrap test, this study further introduced a quasi-Bayesian sensitivity analysis to cross-validate the robustness of this mediation effect across methods. This finding not only provides more focused and robust empirical evidence for understanding the psychological mechanisms underlying depressive symptoms in this specific age group, but also highlights the unique importance of attending to subjective cognitive self-evaluation in promoting health among young-old adults. The following sections will further elucidate the mechanisms underlying these associations, discuss the public health implications of the statistical mediation effect, and objectively outline the study’s limitations and directions for future research.

### 4.1. Associations Among Educational Attainment, Self-Reported Memory Function, and Depressive Symptom Severity in Older Adults Aged 60–74 Years

This study’s findings indicate that higher educational attainment was associated with lower depressive symptom severity among young-old adults, and educational attainment was significantly positively correlated with self-reported memory function, while self-reported memory function was significantly negatively correlated with depressive symptom severity. The negative association between educational attainment and depressive symptoms in older adults (Jeong et al., 2024), as well as the negative association between cognitive function and depressive symptoms, have both been confirmed by numerous large-sample epidemiological studies (Ma et al., 2025; Cristancho et al., 2018; C. Li et al., 2025a). Previous research has shown that older adults with lower educational attainment are more likely to exhibit a “rapidly increasing” trajectory of depressive symptoms (Jeong et al., 2024). An international meta-analysis further confirmed that the negative association between education and depressive symptoms is widespread among older populations, with older adults of lower educational attainment exhibiting significantly higher levels of depressive symptoms in late life (Chang-Quan et al., 2010). Considering patterns of cognitive aging in older adults (Salthouse, 2009), young-old adults aged 60–74 years retain relatively intact cognitive function and have a larger intervention window, a characteristic that may provide more favorable conditions for early intervention targeting the association between education and depressive symptoms.

In this study, educational attainment was moderately positively correlated with self-reported memory function (*r* = 0.241, *p* < 0.001), suggesting a practically meaningful association between educational attainment and self-reported memory function among young-old adults; whereas educational attainment showed a statistically significant but weak negative correlation with depressive symptom severity (*r* = −0.177, *p* < 0.001), indicating that the association between educational attainment and depressive symptom severity among young-old adults is not mediated by a single pathway, and that other potential mediating or moderating factors may also be involved (J. Lee, 2011). In terms of clinical significance, the total effect model in this study showed that for each one-level increase in educational attainment, the CES-D8 depressive symptom severity total score decreased by approximately 0.407 points on average among young-old adults (accounting for about 9% of the CES-D8 standard deviation of 4.483). Although this effect size was highly significant statistically, the absolute score change corresponding to a single-level difference in education is relatively limited in clinical significance, suggesting that the statistical association between education and depressive symptom severity in this population is more likely to operate through long-term, intergenerational cumulative effects rather than short-term, substantial symptom improvement.

The cognitive reserve theory provides a core mechanism to explain the above associations. This theory posits that early-life educational experiences can build “cognitive reserve” by promoting the structural optimization and functional remodeling of brain neural networks (Stern, 2012; Clouston et al., 2020), enhancing an individual’s resilience to neuropathological changes and their ability to maintain function. This reserve can effectively delay age-related cognitive decline and improve an individual’s psychological resilience in coping with negative emotions and life stresses (Le Carret et al., 2003; J. Lee et al., 2018). For young-old adults, the cognitive reserve formed by higher educational attainment may enable more efficient processing of daily information and help maintain normal social cognitive function, thereby being associated with lower levels of core depressive symptoms such as helplessness and hopelessness (Umucu et al., 2022). The average educational attainment score of the young-old participants in this study was only 1.36±1.191, reflecting the generally low educational attainment of this population. Those with lower education, due to insufficient cognitive reserve, are not only more susceptible to declines in self-reported memory function but are also more likely to experience more pronounced depressive symptoms, which also helps explain the significant association between the two (Stern, 2012; Clouston et al., 2020).

### 4.2. The Statistical Mediating Role of Self-Reported Memory Function Between Educational Attainment and Depressive Symptom Severity

The mediation analysis revealed a significant partial mediating effect of self-reported memory function in the relationship between educational attainment and depressive symptom severity among young-old adults aged 60–74 years, accounting for 26.80% of the total effect. The 95% confidence interval for the indirect effect did not include zero, indicating that the mediating effect was statistically stable and significant. These results suggest that approximately one-quarter of the association between educational attainment and depressive symptom severity among young-old adults can be explained through the statistical pathway of self-reported memory function. This effect proportion falls within a similar range to those reported in previous studies among older populations, suggesting a degree of consistency across studies and providing additional support for the robustness of the observed statistical association (James et al., 2021; R. Zhao et al., 2024a).

The multiple memory systems theory posits that episodic memory, as a core component of declarative memory, involves the contextualized recollection of specific times, places, and events, with its neural substrates primarily located in the hippocampus and prefrontal cortex. This memory system is among the earliest and most significantly affected by physiological aging (Martin-Ordas & Easton, 2024; Robertson, 2002). Sustained engagement in education-related cognitive activities may effectively delay age-related atrophy of the hippocampus and prefrontal cortex, whereas individuals with lower educational attainment, due to insufficient cognitive reserve, may exhibit more pronounced atrophy in these brain regions (Vonk et al., 2022), which may be associated with the decline in self-reported memory function. In this study, the positive association between educational attainment and self-reported memory function (B = 0.221, *p* < 0.001) is also consistent with this mechanistic explanation, namely, that higher educational attainment may be associated with better self-reported memory function among young-old adults through the shaping of cognitive reserve (Stern, 2012; Clouston et al., 2020).

The significant negative correlation between self-reported memory function and depressive symptom severity can be interpreted from two perspectives: functional characteristics and psychological mechanisms relevant to the young-old population (James et al., 2021; R. Zhao et al., 2024a). On the one hand, young-old adults aged 60–74 years typically maintain relatively high levels of social engagement and independence in daily living. Self-reported memory function is fundamental for completing everyday tasks such as shopping, social interactions, and household chores. A perceived decline in memory may lead to difficulties in completing daily tasks, thereby triggering feelings of frustration and self-negation (Brailean et al., 2019; C. D. Lee & Foster, 2023), which are associated with depressive symptoms (James et al., 2021); on the other hand, memory function is central to constructing an individual’s sense of self-continuity and social connectedness. Difficulty recalling recent life events may impair young-old adults’ sense of social identity and belonging, increase feelings of loneliness, and thereby be associated with the worsening of depressive symptoms (Aichele & Ghisletta, 2019). Existing research has suggested that memory loss and reduced social activity are factors associated with depressive symptoms among young-old adults, and that this association is more pronounced among groups with lower educational attainment (R. Zhao et al., 2024a), which is consistent with the direction of the findings in this study. This suggests that self-reported memory function represents an important statistical pathway for understanding the association between educational attainment and mental health.

Notably, this study conducted a comprehensive cross-validation of the robustness of the aforementioned mediation pathway through multiple statistical strategies and alternative model specifications. First, a quasi-Bayesian model with 1000 Monte Carlo simulations was employed, and the results showed that the proportion of the indirect effect from the quasi-Bayesian simulation (26.80%) was fully consistent with that of the main model based on the bootstrap method. Second, considering the measurement properties and distributional characteristics of the variables, this study further conducted a Spearman correlation analysis to re-evaluate the associations among the core variables and re-modeled the mediation with educational attainment treated as a categorical variable; the results were highly consistent with the trends of the main analysis (Supplementary Table S1 and S2). In addition, after defining “probable depression” as a binary outcome using CES-D8 ≥ 9, the mediation pathway was re-estimated using logistic regression and modified Poisson regression with robust standard errors; the resulting indirect effect remained significant, and the conclusions were consistent in direction with the main model (Supplementary Table S3). Finally, rigorous model diagnostics indicated no serious multicollinearity (VIF range: 1.023–1.277) or severe influential points (maximum Cook’s distance: 0.006) across the regression models; to address the heteroscedasticity indicated by the tests, this study further re-estimated the models using HC3 robust standard errors, and the significance of the core variables remained unchanged, confirming the reliability of the parameter estimates (Supplementary Table S4). This consistency across statistical methods, variable specifications, and model diagnostics reduces concerns that the findings were driven solely by a particular model specification or estimation method and provides additional support for the robustness of the observed statistical indirect association.

### 4.3. Potential Research Implications and Practical Significance

The findings of this study suggest potential directions for the prevention and intervention of depressive symptoms in the young-old population aged 60–74 years, carry certain practical public health implications, and may also provide insights into cognitive health management and the development of health service systems for older adults. It should be noted that, given the cross-sectional design of this study, the implications discussed below are based on statistical associations rather than causal validation, and their actual applicability requires further examination through prospective or intervention studies.

First, a dual-screening approach involving both memory function and depressive symptoms could be explored to provide a reference for the early identification of depressive symptoms among young-old adults (Aichele & Ghisletta, 2019). Community health service institutions could consider incorporating the single self-reported memory item used in this study, together with the CES-D8 depression scale, into routine health screening programs for young-old adults aged 60–74 years (Brailean et al., 2019; Turvey et al., 1999). Particular attention should be paid to high-risk individuals characterized by low educational attainment combined with poor self-reported memory function to enable early identification and intervention for depressive symptoms through regular screening. This screening model is straightforward and cost-effective, but its actual screening efficacy and applicability still need to be verified by prospective studies.

Second, exploring the design of structured cognitive training programs targeting self-reported memory function, with a particular focus on low-education young-old adults, represents a direction worth examining in future intervention studies. Given that individuals with lower educational attainment are characterized by insufficient cognitive reserve and a higher risk of decline in self-reported memory function, consideration could be given to abandoning traditional, single-mode didactic education models in favor of developing memory training content adapted to the cognitive levels of young-old adults. Specific formats could include daily event retelling training, episodic memory association training, and practical life-skill memory exercises, while combining cognitive training with social group activities to enhance memory capacity and increase social participation. Whether such interventions can effectively reduce depressive symptoms still needs to be verified through randomized controlled trials or longitudinal tracking studies (Aichele & Ghisletta, 2019). Existing intervention studies targeting older women with depression have shown that reminiscence-based cognitive training significantly improves memory function and, to some extent, alleviates depressive and anxiety symptoms, providing preliminary empirical clues for the feasibility of such interventions in older populations (D. Wu et al., 2018); however, whether these conclusions can be generalized to the population in this study remains to be further tested.

Third, integrating cognitive health management for young-old adults into the broader elderly health service system may help alleviate education-related mental health inequalities, although the actual effectiveness of this integrated service model still needs to be evaluated and verified. Increased investment in community-based cognitive health services could be considered, with a particular focus on vulnerable groups such as rural residents and those with lower educational attainment. Practical measures could include establishing community cognitive training rooms, deploying professional cognitive rehabilitation therapists, and organizing public lectures on cognitive health to raise awareness among young-old adults of the importance of cognitive reserve. At the same time, the integration of “cognitive health” and “mental health” services should be promoted, incorporating memory training into intervention plans for individuals at high risk of depressive symptoms and evaluating them jointly, thereby accumulating preliminary evidence for the goal of “using cognitive intervention to promote the prevention and treatment of depressive symptoms” and contributing to the building of active aging (Aichele & Ghisletta, 2019).

Fourth, the findings of this study may offer a reference for the prevention and management of depressive symptoms among older adults in low- and middle-income countries (LMICs). Based on findings from a young-old Chinese population, this study suggests that a statistical-level association pathway exists among educational attainment, self-reported memory function, and depressive symptom severity. Cross-national comparative research indicates that the protective association between education and cognitive function in older adults is evident across countries with varying income levels (Rodriguez et al., 2021), a pattern that closely resembles the characteristics of older populations in many LMICs. Consequently, the screening and intervention strategies proposed in this study may offer valuable insights and references for LMICs with similar national contexts; however, their applicability still needs to be verified in light of local population characteristics.

### 4.4. Limitations and Future Directions

This study demonstrates a degree of methodological rigor: on the one hand, the use of nationally representative data enhances the potential generalizability of the findings; on the other hand, sensitivity analysis based on quasi-Bayesian estimation using 1000 Monte Carlo simulations supported the stability of the effect estimate, thereby reducing, to some extent, concerns about spurious associations and providing statistical support for the study’s conclusions. In addition, by focusing on subjective memory as a core cognitive component and controlling for potential confounding factors such as economic status, this study provides new evidence for understanding the potential cognitive pathway between educational attainment and depressive symptom severity among young-old adults.

Nevertheless, this study still has the following limitations, which should be taken into account when interpreting the results:

First, this study employed a cross-sectional design and therefore cannot support strict causal inferences between variables. In particular, in the field of geriatric mental health, depressive states themselves may lead individuals to develop negative biases regarding their own cognitive abilities (i.e., mood-congruent bias), thereby lowering self-reported memory scores and constituting a potential reverse-causality risk; furthermore, as a subjective cognitive evaluation, self-reported memory function may itself partly reflect a concomitant manifestation of depressive mood rather than a purely independent cognitive construct (Brailean et al., 2019), and the directionality of this relationship warrants cautious interpretation. Although this study, based on “cognitive reserve theory,” constructed and preliminarily validated a positive theoretical pathway of “education–cognition–emotion,” previous longitudinal studies have also suggested that a bidirectional relationship may exist between cognition and depression (Ma et al., 2025), and the cross-sectional data structure of the present study cannot fully rule out this bidirectional influence. Future studies could incorporate longitudinal designs such as cross-lagged panel models to further clarify the causal temporal sequence between self-reported memory and depressive symptoms.

Second, this study did not incorporate early-life socioeconomic indicators such as parental socioeconomic status or childhood family conditions. Educational attainment may, to some extent, serve as a proxy variable for lifelong socioeconomic status (Filigrana et al., 2023; Galobardes et al., 2006); without adjusting for this, it is difficult to determine whether the observed associations reflect the effect of education itself, inherited socioeconomic advantages, or broader life-course social conditions. Constrained by the fact that the 2022 CFPS questionnaire used in this study did not systematically collect information on respondents’ parental early-life socioeconomic status, this study was unable to adjust for this factor, which represents one of its important limitations; future research could supplement this using follow-up questionnaires containing relevant modules.

Third, this study employed complete-case analysis (listwise deletion) to handle missing data and abnormal cases, which may introduce a degree of selection bias. To assess this potential impact, this study compared the characteristics of the 3666 young-old participants included in the analysis with the 2301 excluded cases—comprising 1851 excluded due to missing core variables and 450 excluded due to older age (75–79 years) (see Supplementary Table S5 for details). The results showed statistically significant differences between the two groups in age (t = 41.481, *p* < 0.001) and sex composition (χ^2^ = 32.399, *p* < 0.001)—the excluded group had a higher mean age (71.74 ± 5.25 years vs. 66.40 ± 4.11 years, primarily because it included the separately excluded 75–79-year-old cases) and a slightly higher proportion of females (55.37% vs. 47.76%). This suggests that the data-cleaning process may have affected the representativeness of the age and sex composition to some extent, and an association between the missingness pattern and study outcomes cannot be fully ruled out (Sterne et al., 2009). Given that approximately 450 of the excluded cases resulted from an age exclusion criterion deliberately set at the study design level (rather than from missing data), and that the proportion of missing core variables was assessed as generally manageable, this study adopted the more conservative and transparent complete-case analysis approach rather than multiple imputation; future research could further verify the robustness of the results using multiple imputation or propensity score methods.

Fourth, this study assessed self-reported memory function using a single self-reported item, which, although offering high feasibility and ecological validity in large-scale nationally representative surveys, has limited construct coverage and does not allow for statistical testing of reliability, and, as noted earlier, may be susceptible to mood-congruent bias. Future research could incorporate standardized neuropsychological tests (e.g., the MMSE or MoCA) to improve the comprehensiveness and objectivity of the measurement.

Fifth, this study included only self-reported memory function as the mediating variable and did not incorporate the mediating or moderating roles of other cognitive function dimensions (e.g., executive function, attention) or psychosocial factors (e.g., social support, life events), which may mean that the full set of pathways linking educational attainment and depressive symptoms was not completely captured.

Sixth, although the observed associations were statistically significant and were validated by both the bootstrap and quasi-Bayesian methods, the effect sizes were generally small (e.g., the correlation coefficient between educational attainment and depressive symptom severity was *r* = −0.177), suggesting that educational attainment and self-reported memory function have limited explanatory power for variation in depressive symptom severity, and that the two factors may represent only part of the many contributing influences on depressive symptom severity among young-old adults; the actual public health significance should be interpreted cautiously in light of the effect size. In addition, in the sensitivity analysis treating educational attainment as a categorical variable, the highest educational attainment group (junior college/bachelor’s/master’s degree, *n* = 119) showed reduced precision in effect estimation due to its relatively limited sample size (see Supplementary Table S2), which also suggests that the interpretation of some sensitivity analysis results should take into account the statistical power limitations arising from differences in subgroup sample sizes.

Seventh, the sample in this study was drawn from community-dwelling young-old adults aged 60–74 years in China; the generalizability of the conclusions should therefore be limited to populations with similar characteristics. Their applicability to institutionalized older adults, adults aged 75 years and above, or populations in other countries and regions with healthcare systems substantially different from the context of this study remains to be further verified.

In summary, the main strengths of this study lie in its use of a nationally representative sample and standardized measurement instruments, as well as its cross-validation of the mediation effect using both the bootstrap and quasi-Bayesian statistical methods, which together support the robustness of the results; however, the study is also limited by its cross-sectional design, single-item subjective measurement, potential selection bias, and uncontrolled early-life socioeconomic factors. The findings of this study should therefore be regarded as preliminary evidence of a statistical association among educational attainment, self-reported memory function, and depressive symptoms, rather than a causal conclusion. Future research should employ longitudinal designs, objective cognitive assessments, more comprehensive control of socioeconomic covariates, and the construction of multi-mechanism models to further deepen the understanding of the “education–memory–depression” association, thereby providing a more solid scientific basis for the early prevention and precision intervention of depressive symptoms in older adults.

## 5. Conclusions

This study found that among young-old adults aged 60–74 years, educational attainment was significantly negatively associated with depressive symptom severity, and self-reported memory function played a significant partial statistical mediating role between them—that is, educational attainment was directly associated with lower depressive symptom severity among young-old adults, and may also be indirectly associated with reduced depressive symptom severity through maintaining or enhancing self-reported memory function. It should be noted that, given the cross-sectional design of this study, the above associations should be understood as statistical association pathways rather than confirmed causal relationships.

Based on these findings, strengthening attention to cognitive function among young-old adults—particularly conducting early screening of self-reported memory function among individuals with lower educational attainment and exploring the feasibility of targeted cognitive training—may represent a potential direction for reducing the risk of depressive symptoms in this population in the future; however, its actual effectiveness still requires further examination through prospective or intervention studies. This finding has certain public health implications for promoting mental health among older adults and advancing active aging.

## Figures and Tables

**Table 1:** Descriptive Statistics of Categorical Variables for the Study Participants.

Variable	Count	Percentage
Overall	3666	100%
Sex		
Female	1751	47.76%
Male	1915	52.24%
Residence		
Rural	1873	51.09%
Urban	1793	48.91%
Subjective income level		
Lower	954	26.02%
Higher	2712	73.98%
Self-rated Health		
Poor/Fair	1405	38.33%
Good/Very Good/Excellent	2261	61.67%
Presence of Chronic Disease(s)		
No	2541	69.31%
Yes	1125	30.69%

**Table 2:** Descriptive Statistics of Quantitative Variables for the Study Participants.

	Minimum Value	Maximum Value	Mean Value	Standard Deviation
Age (years)	60	74	66.40	4.105
Educational Attainment (0–4)	0	4	1.36	1.191
Self-Reported Memory Function (0–4)	0	4	1.60	1.301
Depressive Symptom Severity (CES-D8 total score)	0	24	5.80	4.483

**Table 3:** Score Distribution and Descriptive Statistics of Depressive Symptom Items (CES-D 8).

Item	Score Distribution, n (%)				Mean	SD
	0	1	2	3		
D1	1654 (45.12%)	1421 (38.76%)	365 (9.96%)	226 (6.16%)	0.77	0.863
D2	1575 (42.96%)	1191 (32.49%)	487 (13.28%)	413 (11.27%)	0.93	1.004
D3	1558 (42.50%)	1087 (29.65%)	583 (15.90%)	438 (11.95%)	0.97	1.030
D4	1580 (43.10%)	1036 (28.26%)	757 (20.65%)	293 (7.99%)	0.94	0.976
D5	2419 (65.98%)	788 (21.49%)	243 (6.63%)	216 (5.89%)	0.52	0.858
D6	1775 (48.42%)	1017 (27.74%)	641 (17.48%)	233 (6.36%)	0.82	0.938
D7	2289 (62.44%)	1001 (27.30%)	219 (5.97%)	157 (4.28%)	0.52	0.791
D8	2871 (78.31%)	523 (14.27%)	122 (3.33%)	150 (4.09%)	0.33	0.731

Note: D1: I felt depressed. D2: I felt that everything I did was an effort. D3: My sleep was restless. D4: I was happy. D5: I felt lonely. D6: I enjoyed life. D7: I felt sad. D8: I could not get “going”. Items D4 and D6 were reverse-scored.

**Table 4:** Correlation Analysis of Educational Attainment, Depressive Symptom Severity, and Self-Reported Memory Function.

Variable	Depressive Symptom Severity	Educational Attainment	Self-Reported Memory Function
Depressive Symptom Severity	1		
Educational Attainment	−0.177 ***	1	
Self-Reported Memory Function	−0.223 ***	0.241 ***	1

Note: *** *p* < 0.001.

**Table 5:** Mediation model of self-reported memory function between educational attainment and depressive symptom severity among young-old adults aged 60–74.

Dependent Variable	Independent Variable	B	Standard Error	t-Value	*p*-Value	β
Depressive Symptom Severity (Model 1)						
	Age	−0.069	0.017	−4.002	<0.001	−0.063
	Sex (Control group: Female)					
	Male	−0.840	0.143	−5.887	<0.001	−0.094
	Residence (Control Group: Rural)					
	Urban area	−1.042	0.142	−7.342	<0.001	−0.116
	Subjective income (Control Group: Lower)					
	Higher	−1.056	0.157	−6.719	<0.001	−0.103
	Self-rated Health (Control Group: Poor)					
	Better	−2.039	0.15	−13.638	<0.001	−0.221
	Chronic Disease(s) (Control Group: No)					
	Yes	1.117	0.155	7.206	<0.001	0.115
	Educational Attainment	−0.407	0.064	−6.392	<0.001	−0.108
	Self-Reported Memory Function					
	R^2^	0.153				
	Adjust R^2^	0.151				
	F-value	94.211				
Self-Reported Memory Function (Model 2)						
	Age	0.013	0.005	2.524	0.012	0.041
	Sex (Control group: Female)					
	Male	0.087	0.043	2.027	0.043	0.033
	Residence (Control Group: Rural)					
	Urban area	0.288	0.043	6.733	<0.001	0.111
	Subjective income (Control Group: Lower)					
	Higher	0.087	0.047	1.837	0.066	0.029
	Self-rated Health (Control Group: Poor)					
	Better	0.282	0.045	6.254	<0.001	0.105
	Chronic Disease(s) (Control Group: No)					
	Yes	0.014	0.047	0.299	0.765	0.005
	Educational Attainment	0.221	0.019	11.505	<0.001	0.202
	R^2^	0.085				
	Adjust R^2^	0.083				
	F-value	48.499				
Depressive Symptom Severity (Model 3)						
	Age	−0.063	0.017	−3.663	<0.001	−0.057
	Sex (Control group: Female)					
	Male	−0.797	0.141	−5.644	<0.001	−0.089
	Residence (Control Group: Rural)					
	Urban area	−0.899	0.141	−6.369	<0.001	−0.100
	Subjective income (Control Group: Lower)					
	Higher	−1.013	0.156	−6.514	<0.001	−0.099
	Self-rated Health (Control Group: Poor)					
	Better	−1.900	0.149	−12.779	<0.001	−0.206
	Chronic Disease(s) (Control Group: No)					
	Yes	1.124	0.153	7.332	<0.001	0.116
	Educational Attainment	−0.297	0.064	−4.646	<0.001	−0.079
	Self-Reported Memory Function	−0.495	0.054	−9.119	<0.001	−0.143
	R^2^	0.172				
	Adjust R^2^	0.170				
	F-value	94.681				

Note: Model 1 is the total effect model, Model 2 is the prediction model of independent variables on the mediating variable, and Model 3 is the direct effect model after including the mediating variable; all models control for age, sex, residence, subjective income, self-rated health, and chronic disease status.

**Table 6:** Bootstrap Test of Mediating Effect of Self-Reported Memory Function between Educational Attainment and Depressive Symptom Severity (Young-Old Adults Aged 60–74 Years).

Item	Total Effect (c)	Path a	Path b	Mediation Effect Value (a*b)	a*b (Boot SE)	a*b (95%Boot CI)	Direct Effect (c’)	TEST Conclusion
Educational Attainment => Self-Reported Memory Function=> Depressive Symptom Severity (CES-D8 total score)	−0.407	0.221 ***	−0.495 ***	−0.109 ***	0.015	−0.139 to −0.082	−0.297 ***	Partial Statistical Mediating Role

Note: Bootstrap sampling was conducted 5000 times; if the 95% confidence interval does not include 0, the effect is considered significant; *** *p* < 0.001. Path a: Educational Attainment → Self-Reported Memory Function. Path b: Self-Reported Memory Function → Depressive Symptom Severity. Path c: The total effect value of Educational Attainment on the degree of Depressive Symptom Severity. Path c’: The direct effect value of Educational Attainment on the degree of Depressive Symptom Severity.

**Table 7:** Results of the quasi-Bayesian robustness check for the mediation effect.

Type of Effect	Estimate	95% CI	*p* Value
Indirect Effect	−0.109	[−0.140, −0.081]	<0.001
Direct Effect	−0.297	[−0.423, −0.175]	<0.001
Total Effect	−0.407	[−0.531, −0.283]	<0.001
Proportion Mediated	0.268	[0.179, 0.415]	<0.001

Note: The 95% confidence intervals for the estimated effects were obtained using a quasi-Bayesian approximation with 1000 simulations. Given the cross-sectional design, the estimated indirect and direct effects are interpreted only as statistical associations and should not be regarded as evidence of causal effects or causal mediation. All models were adjusted for sex, residence, subjective income, self-rated health, chronic disease status, and age.

## Data Availability

The raw data is publicly available at https://www.isss.pku.edu.cn/cfps/index.htm (accessed on 15 July 2026).

## Supplementary Materials

The following supporting information can be downloaded at: https://www.mdpi.com/article/10.3390/bs16081264/s1, Table S1: Spearman correlation analysis of core variables; Table S2: Sensitivity analysis modeling educational attainment as a categorical variable; Table S3: Sensitivity mediation analysis using probable depression (CES-D8 ≥ 9) as a binary outcome; Table S4: Linear regression model diagnostics and HC3 robust standard errors for the direct effect model; Table S5: Comparison of basic characteristics between the included participants and all cases excluded during sample selection; Figure S1: Residuals versus fitted values plot for the direct effect model (Model 3); Figure S2: Q-Q plot of residuals for the direct effect model (Model 3).
