# Supplementary material for: Association Between Educational Attainment and Depressive Symptom Severity Among Young-Old Adults Aged 60–74 Years: The Mediating Role of Self-Reported Memory Function
Source: Behav Sci (Basel). 2026 Jul 23;16(8):1264. doi: 10.3390/bs16081264 (PMC13509630; doi:10.3390/bs16081264)
Supplement: Supplementary file 1 [file behavsci-16-01264-s001.zip › behavsci-4401471-supplementary.pdf]

Supplementary Table S1. Spearman correlation analysis of core variables

Variables	Educational Attainment	Attainment Self-Reported Memory Function	Depressive Symptom Severity (CES-D8)
Educational Attainment	1.000		
Self-Reported Memory Function	0.239***	1.000	
Depressive Symptom Severity	-0.174***	-0.225***	1.000

Note: *** *P* < 0.001.

Supplementary Table S2. Sensitivity analysis modeling educational attainment as a categorical variable

Variable	n	B	SE	*P-value*	*t-value*	Standardized β
Educational attainment (Reference: No formal schooling and illiterate/semi-illiterate)						
Primary school	772	-0.298	0.192	0.121	-1.549	-0.027
Junior high school	955	-0.717	0.189	<0.001	-3.800	-0.070
Senior high/ Technical secondary /vocational school	612	-0.948	0.221	<0.001	-4.289	-0.079
Junior college/ Bachelor's/Master's degree	119	-0.791	0.411	0.055	-1.922	-0.031
Mediator						
Self-reported memory function	—	-0.496	0.054	<0.001	-9.137	-0.144
Covariates						
Age	—	-0.064	0.017	<0.001	-3.746	-0.059
Sex (Male vs. Female)	—	-0.794	0.142	<0.001	-5.580	-0.088
Residence (Urban vs. Rural)	—	-0.905	0.141	<0.001	-6.397	-0.101
Subjective income level (Higher vs. Lower)	—	-1.020	0.156	<0.001	-6.547	-0.100
Self-rated health (Good vs. Poor)	—	-1.898	0.149	<0.001	-12.762	-0.206
Chronic disease (Yes vs. No)	—	1.123	0.153	<0.001	7.320	0.116

Note: The reference group is "No formal schooling and illiterate/semi-illiterate" (n = 1208). Sample sizes for each educational attainment group are: primary school (n = 772), junior high school (n = 955), senior high/technical secondary/vocational school (n = 612), and junior college/bachelor's/master's degree (n = 119), totaling 3,666, consistent with the main analysis sample size. Sample size distributions for the mediator and covariates are detailed in Table 1 of the main text and are not repeated here. The junior college/bachelor's/master's degree group had a relatively small sample size (n = 119), with a standard error (0.411) markedly higher than that of the other educational attainment groups (0.19–0.22). Consequently, the precision of the effect estimate was lower, and the *P*-value did not reach statistical significance (*P* = 0.055). The reduced precision and non-significant estimate in the highest education group may partly reflect its small sample size. However, a non-linear association cannot be excluded and should be examined in future studies with larger highly educated subgroups.

Supplementary Table S3. Sensitivity mediation analysis using probable depression (CES-D8≥9) as a binary outcome

Pathway	Logistic	Modified Poisson
Path *a*: Educational attainment → Self-reported memory function (B†)	0.221***	0.221***
Path *b*: Self-reported memory function → Probable depression (OR/PR [95% CI])	0.801 [0.750, 0.856]	0.855 [0.817, 0.894]
Total effect *c*: Educational attainment → Probable depression (OR/PR [95% CI]	0.777 [0.720, 0.839]	0.839 [0.795, 0.886]
Direct effect *c'*: Educational attainment → Probable depression (OR/PR [95% CI]	0.811 [0.751, 0.877]	0.866 [0.820, 0.915]
Indirect effect a×b (Exponentiated [95% CI]‡)	0.952 [0.936, 0.967]	0.966 [0.954, 0.977]

Note: † Path a represents the unstandardized coefficient B from the linear regression model (self-reported memory function = educational attainment + covariates), not an odds ratio (OR) or prevalence ratio (PR), and is not directly comparable with the other rows. For the logistic model, OR is interpreted as an odds ratio; for the modified Poisson model, PR is interpreted as a prevalence ratio. ‡ The exponentiated indirect effects (a×b) for both models are based on 5,000 bootstrap resamples; a confidence interval not containing 1 indicates that the indirect association is statistically significant. *** P < 0.001.

Supplementary Table S4. Linear regression model diagnostics and HC3 robust standard errors for the direct effect model

Variable	Original B	Original SE	HC3 Robust SE	HC3 Robust *P*-value
Educational attainment	-0.297	0.064	0.064	<0.001
Self-reported memory function	-0.495	0.054	0.054	<0.001
Age	-0.063	0.017	0.017	<0.001
Sex (Male vs. Female)	-0.797	0.141	0.143	<0.001
Residence (Urban vs. Rural)	-0.899	0.141	0.143	<0.001
Subjective income level (Higher vs. Lower)	-1.013	0.156	0.164	<0.001
Self-rated health (Good vs. Poor)	-1.900	0.149	0.156	<0.001
Chronic disease (Yes vs. No)	1.124	0.153	0.159	<0.001

Note: Variance inflation factors (VIF) ranged from 1.023 to 1.277, indicating no evidence of significant multicollinearity. The maximum Cook's distance was 0.006, indicating no severe influential points. The Breusch-Pagan test indicated the presence of heteroscedasticity ($P <$ 0.001); the model was therefore re-estimated using HC3 robust standard errors. Results showed that the core variables remained highly significant ($P < 0.001$) after applying robust standard errors, demonstrating the robustness of the model's conclusions.

Supplementary Table S5. Comparison of basic characteristics between the included participants and all cases excluded during sample selection

Variables	Included (n = 3666)	Excluded (n=2301)	Statistic	*P*-value
Age, Mean ± SD	66.40 ±4.11	71.74±5.25	t=41.481	<0.001
Sex, n (%)			χ^2 = 32.399	<0.001
Male	1915 (52.24%)	1027 (44.63%)		
Female	1751 (47.76%)	1274 (55.37%)		

Note: The excluded group (n = 2,301) consists of two parts: (1) 1,851 individuals excluded due to missing or abnormal values in core variables (educational attainment, self-reported memory function, depressive symptoms, etc.); and (2) 450 individuals excluded because their age fell outside the 60–74 range in the original data (i.e., aged 75–79 years). Because the excluded group includes this older subgroup, its mean age was significantly higher than that of the included group, which was strictly limited to adults aged 60–74.

Supplementary Figure S1. Residuals versus fitted values plot for the direct effect model (Model 3).

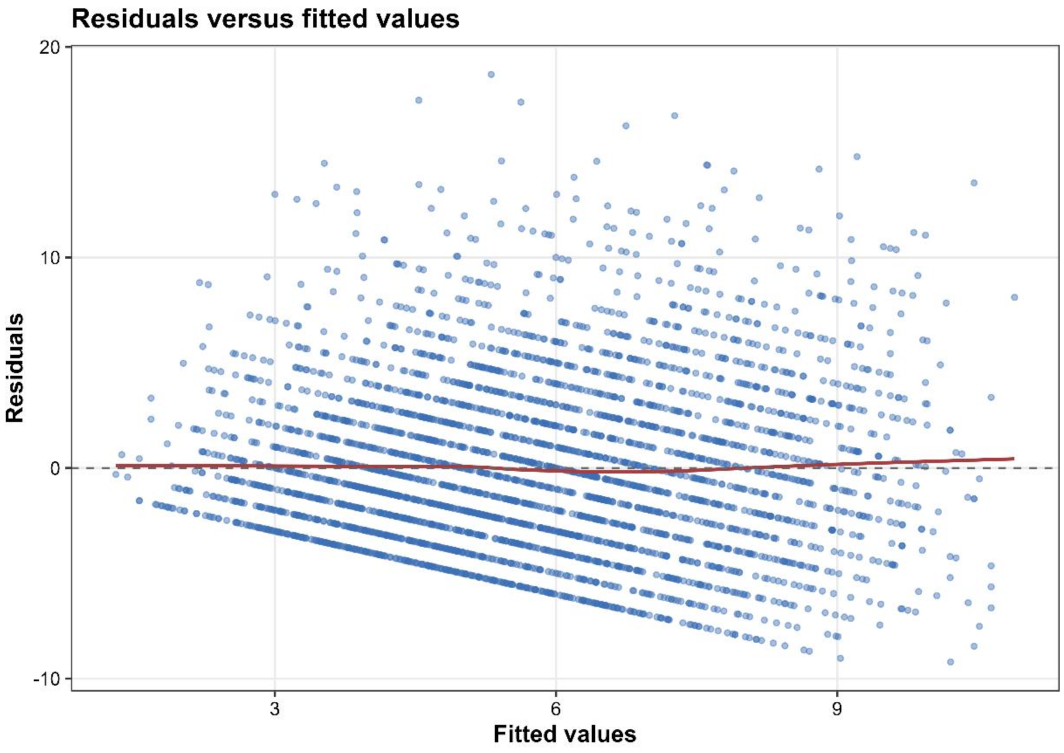

Note: The x-axis represents the model's fitted values, and the y-axis represents the model residuals; the dashed line is the zero reference line, and the red curve is the locally estimated scatterplot smoothing (LOESS) fit line. The striped distribution pattern shown in the figure arises because the outcome variable (CES-D8 total score) is a discrete integer ranging from 0 to 24. The LOESS fit line remains generally close to 0 with no obvious non-linear trend, suggesting that the linear specification of the model is reasonable.

Supplementary Figure S2. Q-Q plot of residuals for the direct effect model (Model 3).

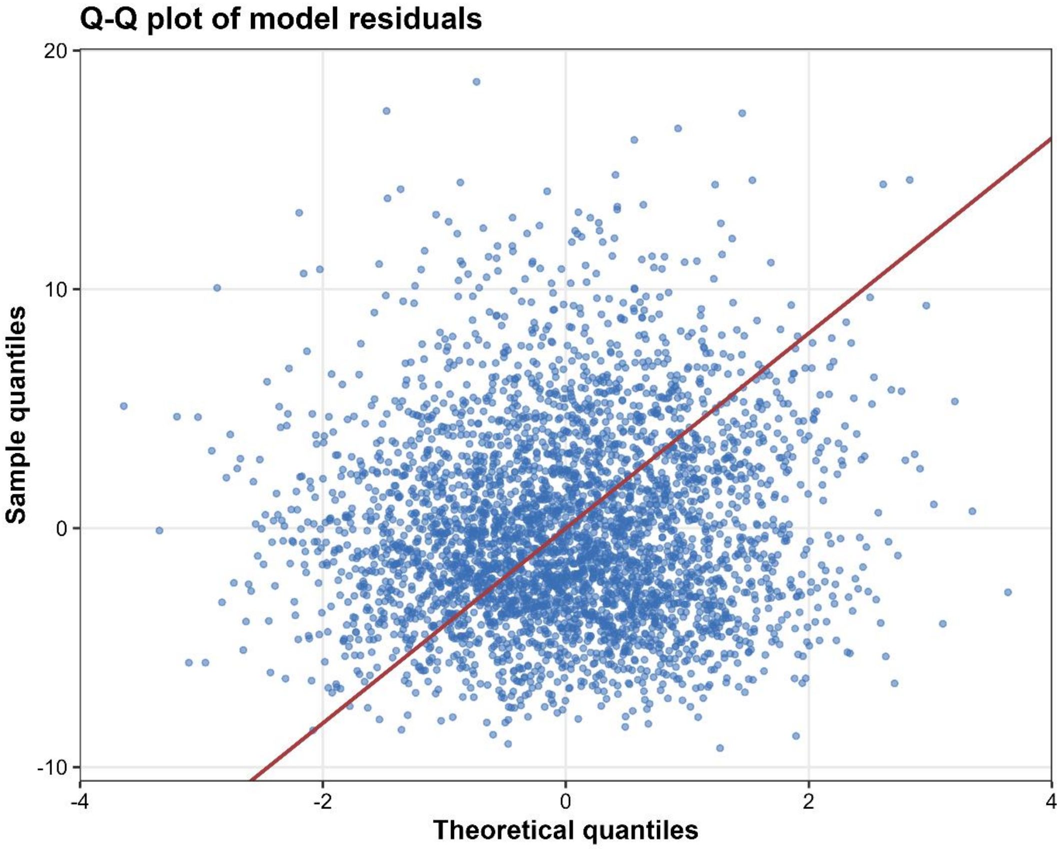

Note: The x-axis represents the theoretical normal quantiles, and the y-axis represents the sample residual quantiles; the red line represents the theoretical normal distribution reference line. Results show some deviation of the residuals at both ends (tails), indicating that the residual distribution does not strictly follow a normal distribution. Because heteroscedasticity was detected separately, the model was re-estimated using HC3 robust standard errors (see Supplementary Table S4).
